# The Influence Mechanism of High School English Grammar Science, Technology, Engineering, Art, and Mathematics Teaching Model on High School Students’ Learning Psychological Motivation

**DOI:** 10.3389/fpsyg.2022.917167

**Published:** 2022-07-25

**Authors:** Hong Lin

**Affiliations:** College of Liberal Arts, De La Salle University, Dasmariñas, Philippines

**Keywords:** high school English grammar, STEAM education, traditional teaching mode, psychological motivation, innovation, practice

## Abstract

This study aims to improve the effectiveness of English grammar teaching in high school. Firstly, the Science, Technology, Engineering, Art, and Mathematics (STEAM) educational model is comprehensively discussed. Then, the current situation and difficulties of English grammar teaching in high school are analyzed. Finally, based on the traditional and the STEAM teaching mode, a comprehensive study on the psychological motivation of students is carried out in high school English grammar teaching. The traditional teaching model had little effect on students’ psychological motivation in the process of English grammar teaching. Therefore, students’ satisfaction with English grammar teaching under the traditional teaching model is very low, especially in innovation and practice. The STEAM teaching model has greatly improved the psychological motivation of high school students in the English grammar teaching process. The overall satisfaction rate of the students with the English grammar teaching in the STEAM teaching model is 70%. This teaching model can greatly improve students’ requirements for innovation and practice. This study not only provides a reference for the improvement of English teaching in high schools but also contributes to the reform and development of education.

## Introduction

Education is an eternal development project of society because the development of education can drive the development of the whole society. Therefore, human research on education is very necessary (Guci et al., 2021). English and teaching, as one of the main teaching subjects in China, is imperative to improve its teaching quality. In improving the quality of English teaching, English grammar teaching is the main method. To a certain extent, improving students’ learning effect of English grammar needs to rely on the psychological level, thereby strengthening their interest in learning and promoting the comprehensive learning effect (Aniuranti et al., 2021). Although the comprehensive application of this aspect is not perfect in the current society, many studies have provided a reference for it.

Huang et al. (2020) pointed out that Science, Technology, Engineering, Art, and Mathematics (STEAM) education is based on mathematics. They interpret science and technology from the perspective of engineering and art, integrate different types of subjects with an interdisciplinary concept, and provide excellent human resource support for the development of modern society. The application innovation of STEAM education needs to rely on the new generation of information technologies such as the Internet of Things and cloud computing to create an intelligent education information ecosystem. The system architecture includes “one center, two mechanisms, three resource repositories, four technologies, and users.” At present, the application of STEAM education exists in the environment of lack of implementation plans, shortage of educational resources, lack of professional teachers and training mechanisms, and insufficient hardware equipment and funds (Huang et al., 2020). Perales and Aróstegui (2021) included arts and humanities in the core of the curriculum and science and technology. They analyzed the emergence of the STEAM movement, its implementation in the classroom, and its social, economic, and educational consequences. The conclusion is that, without ignoring the economic rationale of education, it is necessary to embrace further a more social and democratic view of schooling, an attempt to use this philosophy to turn education in a more humanistic direction. They provide a comprehensive education for new generations without ignoring the scientific level while responding to the social and economic needs of today’s world (Perales and Aróstegui, 2021). Rodríguez-Nieto and Alsina (2022) pointed out that under the background of quality education focusing on cultivating language communicative competence, grammar teaching should be combined with communicative language courses, and adhere to the principles of practicality, pertinence, differentiation, and progressiveness. They indicated that teachers should pay attention to the use of communicative methods and traditional methods together, focus appropriately, highlight key teaching requirements, and correct the status of grammar teaching in the entire English teaching (Rodríguez-Nieto and Alsina, 2022). Andriani et al. (2021) pointed out that according to the actual application of situational teaching strategies, students’ interest in learning has been continuously enhanced. Through the vivid, concrete, and vivid display of real-life scenes, students can deeply feel the fun of learning. It has an important impact on improving teaching efficiency (Andriani et al., 2021). One important distinction between traditional and communicative methods is motivation. Xie et al. (2022) pointed out that learning motivation is one of the important factors for the success of foreign language learning and determines the active participation of learners in the process of English learning. However, students’ motivation to learn is not static. It will gradually decline due to the influence of some external or internal factors, which is the so-called “de-motivation” or “motivation weakening.” Questionnaire and interview methods have been used. The three main factors that influence the impairment of motivation in high school were explored (Xie et al., 2022). Shen et al. (2019) pointed out that motivation is a very complex and extensive psychological phenomenon, and motivational factors are included in all psychological activities and psychological phenomena of human beings. Learning activities are one of the most common and important activities in a person’s life. In the long-term educational practice, people increasingly feel that motivation plays a huge role in promoting, motivating, and guiding learning activities. Concern and research on learning motivation have always been an important topics in education and teaching activities. In high school English teaching, learning motivation is also a key factor related to success or failure (Shen et al., 2019).

To sum up, firstly, the STEAM education model is discussed. Secondly, the present situation and connotation of English grammar teaching in senior high school are explored. Finally, the traditional high school English grammar teaching and the English grammar teaching effect under STEAM education are compared, and the influence of the STEAM education model on the psychological mechanism of high school students is studied. The innovation lies in introducing the motivational factors in psychology and comprehensive analysis of students’ English learning according to different classifications. This study provides a reference for improving the effect of English grammar teaching in high school and contributes to improving the quality of social education.

## Research Theories and Methods

### Science, Technology, Engineering, Art, and Mathematics Education

Science, Technology, Engineering, Mathematics (STEM) education originated in the United States. It is a teaching model with links to science, technology, engineering, and mathematics (Marín-Marín et al., 2021). However, STEM education does not simply superimpose the knowledge of science, technology, engineering, and mathematics, but rather integrates and extends the knowledge of these four disciplines, so that the knowledge of these four disciplines forms an organic whole. In the educational process, this educational method can enhance students’ innovative ability and ability to manage and apply multidisciplinary knowledge, and cultivate students’ multi-faceted problem-solving ability (Hsiao and Su, 2021). The emergence of STEM education has not only changed the traditional teaching concept and teaching method to a great extent, but also provided a technical foundation for people to explore a new teaching mode. STEM education also integrates art into it in the later development to form STEAM education. Therefore, STEAM education also includes knowledge about art. Art in a broad sense includes humanities and arts such as society, fine arts, language, and music (Kwan and Wong, 2021). STEAM education has been greatly extended and developed based on STEM education, and a new education model has been formed. It integrates technology and engineering education and arts and humanities education to drive innovation and development in technology-related teaching (Juškevičienė et al., 2021).

**FIGURE 1:**
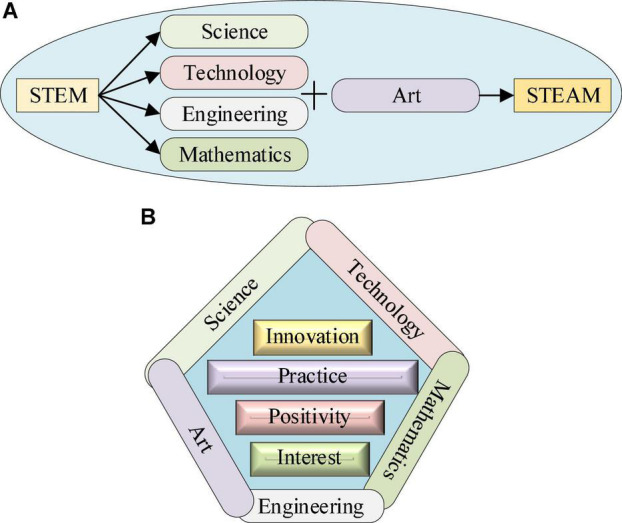
The development form and comprehensive concept of the Science, Technology, Engineering, Art, and Mathematics (STEAM) teaching model (Panel **A** is the development form, Panel **B** is the comprehensive concept).

Science, Technology, Engineering, Art, and Mathematics education also structures science, technology, engineering, mathematics, and the arts to a certain extent. This arrangement is arranged in the spatial structure and plays a role in people’s world transformation and knowledge analysis (Morales et al., 2021). In STEAM education, the world is cognition through scientific knowledge, the world is transformed through engineering and technology, the world is rendered through art, and engineering, technology, science, and art are comprehensively analyzed and expanded through mathematics (López et al., 2021). The development form of STEAM education and its comprehensive concept are shown in Figure 1.

In Figure 1, STEAM education has accumulated many characteristics in its development, including interdisciplinary, collaborative, contextual, design, and experiential (Dolgopolovas and Dagienë, 2021). The interdisciplinary nature of STEAM education is one of its most important features. This feature means that STEAM education is not a separate discipline, but an integration of multiple disciplines, including science, engineering, technology, art, and mathematics. In the education process, a subject education integrates the knowledge of other subjects throughout the education, so that students can master the comprehensive application of multiple subjects to cultivate students’ ability to integrate multiple subjects for innovation and interdisciplinary thinking (Tanabashi, 2021). The situational nature of STEAM education is a more important feature. Because context is an important and meaningful part of the STEAM education process (Hunter-Doniger, 2021). STEAM education emphasizes that educational content and questions come from real life situations. In the process of solving the problem, a more realistic method is selected based on the actual situation, so that students can experience the real life in the learning process, apply what they have learned, and finally get the recognition of the society. The collaborative nature of STEAM education means that students need to communicate and discuss with other students and teachers to a certain extent during the learning process, and then the writing system between students. Students solve learning tasks in small groups, and grow through discussions and joint exploration. Learning in a collaborative way can complement each other’s strengths and weaknesses, stimulate students’ interest in learning, and improve students’ learning effects to a greater extent (Singh, 2021). The experiential nature of STEAM education means that STEAM education pays more attention to the teaching process in the education process, rather than focusing more on the recognition of the final grades as in the traditional teaching mode. STEAM education needs to emphasize the importance of hands-on practice and active participation in the learning process for students in teaching. While obtaining learning results, one can also experience the huge energy contained in the learning process, improve the effect of the teaching process and the comprehensive experience of students’ learning (Lee, 2021). The design of STEAM education means that STEAM education will conduct a certain review of students’ teaching achievements in the final stage of teaching. It emphasizes the importance of students’ own design, improves students’ practical ability and innovation ability, and tests students’ mastery and application of learning content (Vries, 2021). Firstly, students who are taught traditional English grammar are given a questionnaire, students who are taught using traditional English grammar are given a questionnaire. The data obtained from the survey is analyzed. Then, the STEAM education model is used to teach students for a period. Through teaching, students can conduct another questionnaire survey and analyze the data obtained from the survey. Finally, the data obtained from the two questionnaires are compared. The influence mechanism of STEAM education mode on the psychological motivation of high school students has been comprehensively studied. The experiment analyzes the comprehensive influence of STEAM education mode on English grammar teaching in senior high school.

### High School English Grammar Teaching

English grammar teaching in high school is a very important part of English teaching. English teaching in China starts from primary school. The general English teaching content in elementary school is simple word spelling and sentence reading. The content of English teaching in junior high school is generally word memory, sentence reading and writing, and simple grammar teaching. In high school, students need to be taught in-depth grammar and difficult words. Therefore, it is very important to study English teaching in high school. The traditional high school English grammar teaching is mainly through the teacher’s explanation and teaching. Students learn by rote memorization and grammar in sentences (Purwani, 2021). This teaching method not only greatly reduces students’ communication opportunities, making it more difficult for students to understand the content of English learning, but also reduces students’ innovative ability, resulting in students being unable to add their own ideas in the process of English learning. Some teachers think that English learning requires in-depth teaching of students in communication, reducing the teaching of English grammar. Innovative research on English grammar teaching is imperative. Therefore, the main shortcomings of current high school English grammar teaching need to be further explored (Nguyen, 2021).

According to the current knowledge of English grammar teaching, the problems of English grammar teaching in high school are lack of interest, lack of flexibility, lack of practicality, and lack of creativity. The lack of interest is caused by the way teachers teach in the teaching process. Psychologists believe that interest in learning is the main driving force that drives a person to learn and perceive the world (Pham and Tran, 2021). Therefore, in high school English teaching, teachers should improve their own teaching methods and carry out English grammar teaching on the premise of fully mobilizing students’ interest in learning. This improvement can be done in two ways (Ozkan and Umdu, 2021). Firstly, students need to be taught in accordance with their aptitude, that is, through the investigation of students’ psychology and daily study habits, students should be taught English grammar according to their interests. This method is suitable for teaching most subjects. Secondly, active interaction in the classroom is also very important. That is, teachers can make students maintain a high learning state in the learning process through active interaction in the classroom, and make students actively participate in the teaching process by asking questions. Such an approach can improve students’ mastery of teaching content (Arindora et al., 2021). Lack of flexibility means that the current knowledge of the English grammar teaching process teaches students the basic learning content, but does not teach students how to use the teaching content. Therefore, teachers need to carry out flexible teaching, so that students can learn the basic content of English grammar while also learning the skills of listening, speaking, reading, and writing (Torres and Santos, 2021). The main reasons include two aspects: firstly, teachers are too rigid in teaching material content in the teaching process, and do not carry out teaching according to the actual situation. Secondly, teachers are too limited in the analysis of lexical and grammar, resulting in a lack of skill and fun in the teaching process (Li, 2022). Lack of practicality, that is, teachers lack the actual teaching content in the teaching process. High school English grammar belongs to teaching grammar, and its purpose is to let students know the basic rules of English until they are communicatively practiced. Therefore, in the teaching of English grammar in high school, practice should be taken as the overall program, and skill training should be the core of teaching (Tavakoli, 2021). Lack of creativity means that the current high school English grammar teaching methods and methods are too single. For students, this teaching process lacks novelty, and the teaching process is too dull. Therefore, students’ interest in learning will also be greatly reduced (Srisuruk and Siri, 2021).

**FIGURE 2:**
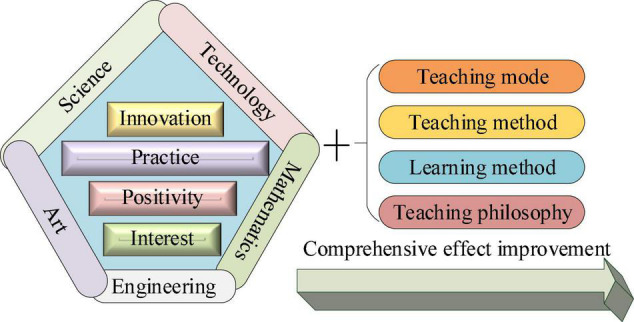
The impact of Science, Technology, Engineering, Art, and Mathematics (STEAM) teaching mode on English grammar teaching in high school.

Through these analyses, the current situation of English teaching in senior high schools lacks innovation, skills, and practical teaching. The STEAM teaching model is used to improve English grammar teaching in high school, which can greatly enhance its teaching method and students’ interest in learning (Rijt and Coppen, 2021). The high school English teaching mode under the STEAM education mode is shown in Figure 2.

In Figure 2, the STEAM teaching model is used to strengthen the English grammar teaching process in high school. This model can not only improve its fundamental teaching methods, but also comprehensively improve students’ learning effects by enhancing students’ interest in learning and learning ability. As an important English teaching project, high school English grammar teaching is very necessary for its research. Additionally, this study will also provide reference for the improvement of its teaching model (Çiftci and Özcan, 2021).

### Research Design

The psychological motivation of high school students is more complex. High school students have deepened their cognition of the outside world, which leads to their complex psychology. They have more subjects and heavier learning tasks. The combined effect of these two aspects has deepened the psychological pressure on students (Chen et al., 2019). In the current high school teaching, teachers usually ignore the psychological changes and psychological pressure of students, which makes teachers’ teaching methods unacceptable to students to a certain extent. The psychological characteristics of high school students mainly include age, gender, and individual differences (Deng et al., 2019).

Age difference means that students will have different psychological changes with age in the learning process. Gender difference refers to the different learning psychology between boys and girls in high school. Individual differences mainly include differences in students’ abilities, temperaments, characters, and interests. The influencing factors of learning psychology mainly include learning motivation, personality, and the social environment in which students live (Zhong et al., 2020). Learning motivation refers to the internal psychological state of students. Higher learning motivation can stimulate students’ learning activities. Students’ personality factors mainly include students’ cognitive style, psychological anxiety, students’ cognition of their own behavior, and their ability to control themselves. Collective and social factors mainly include classroom atmosphere, teachers’ leadership style and role, competition and cooperation process, and social gender roles (Liu et al., 2021).

This study uses a questionnaire survey to conduct psychological research on high school students. It explores the influencing factors of the application of the STEAM education model to high school English grammar teaching. In the process of conducting the questionnaire survey, this study mainly conducts research on the students of a middle school. Firstly, it classifies the students by gender, study subject type, and grade. Secondly, through multi-angle research, students’ learning conditions are comprehensively evaluated, reflecting the specific role of STEAM teaching methods on students. Finally, the students’ satisfaction with the teaching model is evaluated by analyzing the research results. The basic information of the research object is shown in Table 1.

**TABLE 1:** Study groups and their basic conditions.

Classification	Sub-categories	Number of people	Proportion (%)
Gender	Male	233	46.6
	Female	267	53.4
Student	Liberal Arts student	178	35.6
	Science student	168	33.6
	Art student	154	30.8
Grade	Senior 1	165	33.0
	Senior 2	173	34.6
	Senior 3	162	32.4

In Table 1, the selected students are classified according to their psychological characteristics so that their psychological changes of the students can be analyzed comprehensively. According to the comparison between the traditional English grammar teaching method and the STEAM education mode, the psychological changes of students are studied. Five hundred questionnaires are distributed for the first time (for students who received traditional English teaching), and 487 questionnaires are recovered. For the second time, 500 questionnaires (for students who received the STEAM education model) are distributed, and 491 are recovered. Therefore, the designed questionnaire is valid.

This study makes statistics on the questionnaire data received and analyzes it with the Analysis of Variance (ANOVA) method. By comparing students’ educational results before and after, the specific changes of middle school students in the STEAM education environment are determined to analyze the role of the STEAM education model.

## Research Results of High School Students’ Learning Psychology

### Traditional English Grammar Teaching and Motivation

**FIGURE 3:**
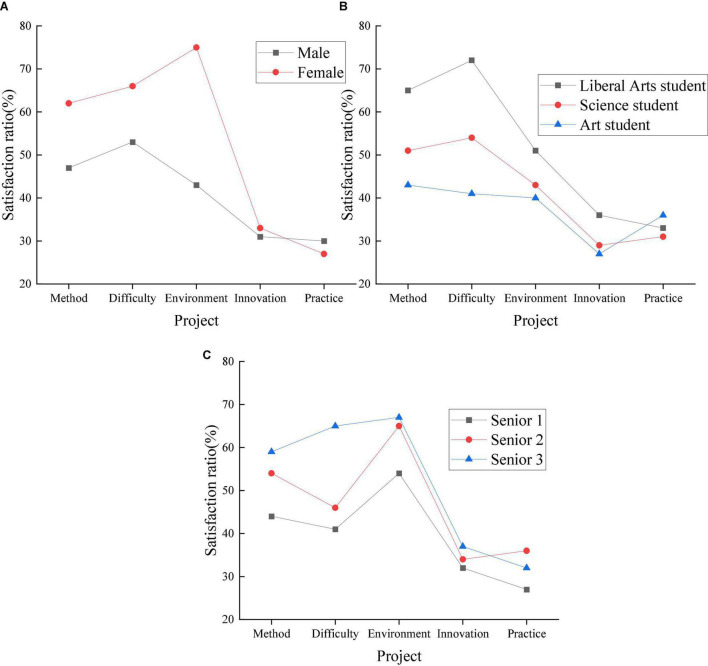
High school students’ satisfaction with traditional English grammar teaching (Panel **A:** gender classification, Panel **B:** learning direction classification, Panel **C:** grade classification).

Firstly, students are surveyed on English grammar. The specific reactions of traditional English grammar teaching methods, teaching contents and teaching effects are analyzed through the investigation and study of students’ overall psychological performance of English teaching. Thus, the rationality of the grouping is verified. The specific psychological mechanism of students on traditional English grammar is shown in Figure 3.

In Figure 3, in the three categories, different students showed great differences. In different gender categories, girls are more satisfied with traditional teaching methods. Girls have the highest percentage of satisfaction with the learning environment, around 76%; the lowest percentage of satisfaction is teaching practice, around 28%. Boys have the highest percentage of satisfaction with the difficulty of teaching content, around 55%, and the lowest percentage of satisfaction with teaching practice, around 30%. Both boys and girls are less satisfied with teaching innovation and teaching practice. In classifying students’ learning direction, liberal arts students have the highest satisfaction with traditional teaching methods, followed by science students, and art students the lowest. Liberal arts students have the highest percentage of satisfaction with the difficulty of teaching content, about 75%; science students have a higher percentage of satisfaction with teaching difficulty, around 55%; art students have a higher percentage of satisfaction with teaching methods, around 43%. The three types of students have the lowest satisfaction with teaching innovation and teaching practice, around 25–38%. In the grade classification, the three types of students have the highest satisfaction with the learning environment, around 50–70%; the lowest satisfaction with teaching innovation and teaching practice, around 25–39%. Students’ satisfaction with the teaching innovation and teaching practice of traditional teaching is relatively low. Therefore, pedagogy needs more research to provide a reference for this field, promote the reform of the traditional teaching model, and improve the comprehensive teaching effect on society.

### The Influence of the Science, Technology, Engineering, Art, and Mathematics Education Model on Motivation

**FIGURE 4:**
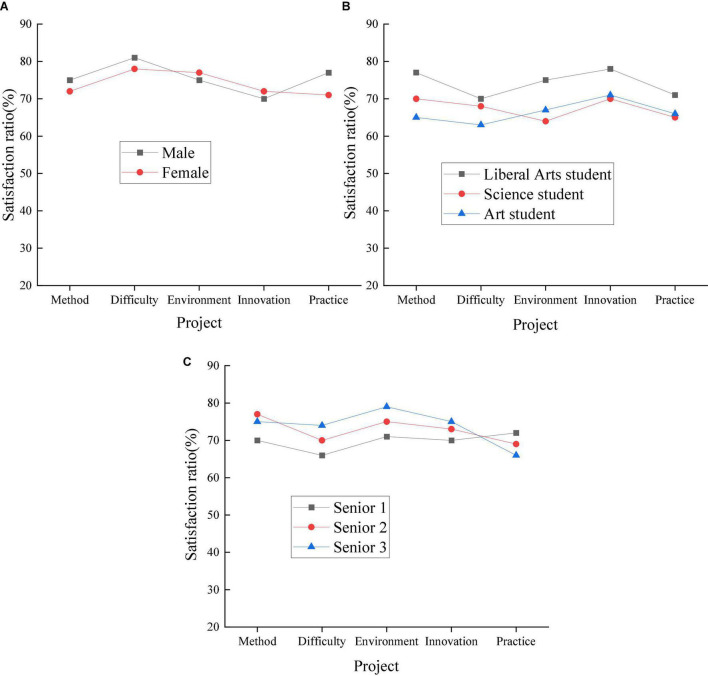
Influence mechanism of Science, Technology, Engineering, Art, and Mathematics (STEAM) education model (Panel **A:** gender classification, Panel **B:** learning direction classification, Panel **C:** grade classification).

The STEAM education model is used to conduct research on English grammar teaching in high schools. The teaching mode plays a great role in innovation, practice, and comprehensive application. Therefore, the research can provide important reference for English grammar teaching in high school, and provide a certain impetus for the improvement of traditional teaching mode. Figure 4 shows the results of STEAM education’s teaching and research on high school students.

**FIGURE 5:**
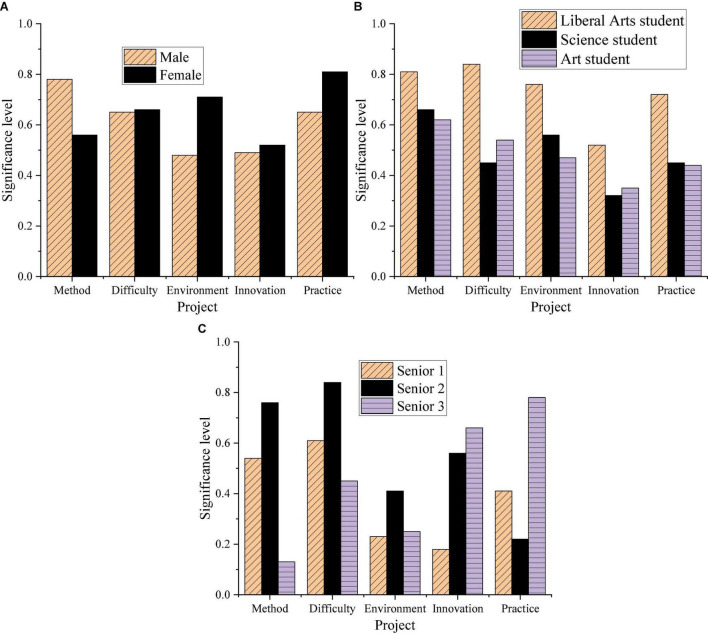
Analysis of the effect of Science, Technology, Engineering, Art, and Mathematics (STEAM) teaching (Panel **A:** gender classification, Panel **B:** learning direction classification, Panel **C:** grade classification).

In Figure 4, under the STEAM education model, the gap between different categories of high school students is reduced; the maximum difference between boys and girls is about 5%; the maximum difference between students of different majors is about 10%; the maximum difference between students of different grades around 7%. STEAM has a great influence on the psychological mechanism among students of different categories and can promote the overall learning and development of students. Additionally, the gap between students in different research projects has also been greatly reduced after STEAM teaching. Compared with the traditional teaching model, students’ satisfaction with teaching innovation and teaching practice has also been improved after STEAM teaching. The ANOVA analysis results of the student survey data under different teaching modes are shown in Figure 5.

In Figure 5, the learning status of high school students in the traditional teaching mode and the STEAM teaching model is compared. Under the STEAM teaching mode, students’ learning situation has changed significantly.

## Discussion

This study aims to improve the comprehensive effect of applied grammar teaching and promote the development of English grammar teaching in senior high schools. The STEAM teaching model is used to analyze the English teaching effect on high school students to improve the students’ learning situation and promote the reform of the teaching mode through research. Firstly, through different classifications, students are surveyed on traditional teaching models. In the three categories, different students showed great differences. Among different gender categories, girls are more satisfied with traditional teaching methods. Girls have the highest percentage of satisfaction with the learning environment and the lowest percentage of satisfaction with teaching practice; boys have the highest percentage of satisfaction with the difficulty of teaching content and the lowest percentage of satisfaction with teaching practice. Students of different genders are less satisfied with teaching innovation and teaching practice. Both liberal arts and science students are more satisfied with the difficulty of teaching; art students are more satisfied with the teaching method. Students are the least satisfied with both teaching innovation and teaching practice. Across the grades, students are the most satisfied with the learning environment; the least satisfied with teaching innovation and teaching practice. Students’ satisfaction with the teaching innovation and teaching practice of traditional teaching is relatively low. Secondly, in investigating students’ learning status under the STEAM education model through different classifications, the gap between students is reduced. STEAM has a great influence on the psychological mechanism of high school students and can promote the overall development of students. Compared with the traditional teaching mode, after the application of STEAM teaching, students’ satisfaction with teaching innovation and teaching practice has been significantly improved. Finally, the learning status of high school students under the traditional and the STEAM teaching mode is compared, and the students’ learning status has changed significantly under the STEAM teaching mode. In the current educational environment, the STEAM teaching model can effectively promote the development of teaching reform. Students are investigated more directly than Mahmudovna et al. (2022). The research methods used are more specific (Mahmudovna et al., 2022). Therefore, the purpose of the study is clearer and more targeted, and applicable to the optimization of English grammar teaching.

## Conclusion

This study studies the psychological mechanism of students in the process of high school English grammar teaching based on the STEAM education model and analyzes the students’ satisfaction with the teaching process between the traditional research model and the STEAM education model. Firstly, English grammar teaching in senior high school is comprehensively discussed. Then, the STEAM education model is analyzed. Finally, based on the STEAM English grammar teaching model, the comprehensive satisfaction of students is investigated. The results show that under the traditional teaching model, students’ overall satisfaction with the teaching process is low, and students’ overall satisfaction with teaching innovation and teaching practice is the lowest. This study surveyed students after adopting the STEAM education model, and the students’ satisfaction with all aspects of the teaching process has been greatly improved. Students’ overall satisfaction is relatively high, and the overall satisfaction rate is above 70%. Students’ satisfaction with teaching innovation and teaching practice has been improved the most, and high school English grammar teaching has high requirements in terms of innovation and practice. Therefore, the STEAM teaching mode plays an important role in improving students’ learning psychology. STEAM teaching mode can significantly improve students’ learning situations. This study provides the influencing factors of STEAM teaching mode on the psychological mechanism of high school students, the specific factors provided are less involved. Therefore, future research will increase the influencing factors of psychological mechanisms to improve the STEAM teaching model further.

## Data Availability Statement

The raw data supporting the conclusions of this article will be made available by the authors, without undue reservation.

## Ethics Statement

The studies involving human participants were reviewed and approved by De La Salle University Ethics Committee. The patients/participants provided their written informed consent to participate in this study. Written informed consent was obtained from the individual(s) for the publication of any potentially identifiable images or data included in this article.

## Author Contributions

The author confirms being the sole contributor of this work and has approved it for publication.

## Conflict of Interest

The author declares that the research was conducted in the absence of any commercial or financial relationships that could be construed as a potential conflict of interest.

## Publisher’s Note

All claims expressed in this article are solely those of the authors and do not necessarily represent those of their affiliated organizations, or those of the publisher, the editors and the reviewers. Any product that may be evaluated in this article, or claim that may be made by its manufacturer, is not guaranteed or endorsed by the publisher.
