# Supplementary material for: The Influence Mechanism of High School English Grammar Science, Technology, Engineering, Art, and Mathematics Teaching Model on High School Students’ Learning Psychological Motivation
Source: Front Psychol. 2022 Jul 25;13:917167. doi: 10.3389/fpsyg.2022.917167 (PMC9358022; doi:10.3389/fpsyg.2022.917167)
Supplement: Supplementary file 1 [file Data_Sheet_1.docx]

**Appendix 1**

Questionnaire

Traditional English grammar teaching satisfaction questionnaire (1-5 are: very dissatisfied, not satisfied, somewhat satisfied, satisfied, very satisfied):

1. Satisfaction with traditional English grammar teaching methods:

◻1 ◻2 ◻3 ◻4 ◻5

2. Satisfaction with the difficulty of traditional English grammar teaching:

◻1 ◻2 ◻3 ◻4 ◻5

3. Satisfaction with the traditional English grammar teaching environment:

◻1 ◻2 ◻3 ◻4 ◻5

4. Satisfaction with the degree of innovation in traditional English grammar teaching:

◻1 ◻2 ◻3 ◻4 ◻5

5. Satisfaction with traditional English grammar teaching and practice methods:

◻1 ◻2 ◻3 ◻4 ◻5

6. Comprehensive satisfaction with the traditional teaching mode:

◻1 ◻2 ◻3 ◻4 ◻5

7. Suggestions for improving the traditional teaching mode:

STEAM English Grammar Teaching Satisfaction Questionnaire:

1. Satisfaction with STEAM English grammar teaching method:

◻1 ◻2 ◻3 ◻4 ◻5

2. Satisfaction with the difficulty of STEAM English grammar teaching:

◻1 ◻2 ◻3 ◻4 ◻5

3. Satisfaction with the STEAM English grammar teaching environment:

◻1 ◻2 ◻3 ◻4 ◻5

4. Satisfaction with the degree of innovation in STEAM English grammar teaching:

◻1 ◻2 ◻3 ◻4 ◻5

5. Satisfaction with STEAM English grammar teaching practice:

◻1 ◻2 ◻3 ◻4 ◻5

6. Comprehensive satisfaction with the STEAM teaching model:

◻1 ◻2 ◻3 ◻4 ◻5

7. Suggestions for improving the STEAM teaching model:
